# The social anatomy of AI anxiety: gender, generations, and technological exposure

**DOI:** 10.3389/fpsyt.2025.1641546

**Published:** 2025-11-21

**Authors:** Naciye Güliz Uğur, Faruk Dursun

**Affiliations:** Department of Management Information Systems, Sakarya University, Business School, Sakarya, Türkiye

**Keywords:** AI anxiety, technoparanoia, sociotechnical risk, measurement validation, effect size, hierarchical regression, public policy, AI literacy

## Abstract

**Introduction:**

Public anxiety surrounding artificial intelligence (AI) carries significant clinical, educational, and policy implications. However, evidence regarding the multidimensional structure of AI-related anxiety and its demographic and experiential correlates remains fragmented. This study synthesizes validated measures into a coherent framework to examine how psychological and sociodemographic factors shape AI-related anxieties.

**Method:**

A cross-sectional survey of adults (N = 1,151) assessed nine dimensions of AI-related anxiety --general AI anxiety, technoparanoia, technophobia, AI interaction anxiety, job-replacement anxiety, sociotechnical blindness, cybernetic-revolt fear, technology self-efficacy, and AI learning orientation --adapted from established scales. Dimensionality was evaluated using common-factor exploratory factor analysis (principal axis factoring, Promax rotation; KMO = .89; Bartlett's p < .001), supported by parallel analysis and scree inspection. A 70/30 hold-out confirmatory factor analysis assessed structural validity. Reliability (Cronbach's α, McDonald's ω), composite reliability (CR), and average variance extracted (AVE) were calculated to examine internal consistency and convergent validity, while discriminant validity used the Fornell -Larcker and HTMT criteria. Group differences were tested using t-tests and ANOVA with Holm -Bonferroni correction and effect sizes. Hierarchical regression models controlled for age, gender, marital status, employment, and AI-use status.

**Results:**

The nine-factor structure was supported (64.17% variance explained). CFA indicated good fit (CFI = .943, TLI = .936, RMSEA = .045 [90% CI .041 -.049], SRMR = .046). All scales demonstrated strong reliability (α, ω ≥ .80), convergent validity (CR ≥ .83; AVE ≥ .51), and discriminant validity. After correction for multiple comparisons, gender differences remained for technoparanoia, AI learning orientation, and AI interaction anxiety (small effects, Cohen's d ≈ .18 -.21). AI users exhibited higher general AI anxiety, technoparanoia, and sociotechnical blindness (d ≈ .17 -.29). Age-group differences were non-significant. Hierarchical regression showed that sociotechnical blindness and technoparanoia were the strongest positive predictors of general AI anxiety, while technology self-efficacy and AI learning orientation were negative predictors.

**Discussion:**

AI-related anxiety is a reliable and multidimensional construct, driven more by psychological dispositions and technology experience than by demographic characteristics. The findings suggest actionable pathways for mitigating anxiety, including targeted AI literacy initiatives, strengthening self-efficacy, and transparent communication regarding sociotechnical impacts. These interventions may support informed and equitable AI integration across clinical, educational, and policy contexts.

## Introduction

Artificial Intelligence (AI), once the domain of science fiction, has rapidly transitioned into an integral component of contemporary society, influencing diverse areas including employment, healthcare, education, and communication. Globally, AI is projected to exceed $190 billion globally, underscoring its central role in technological innovation and economic growth (1). However, this swift integration has generated significant public apprehension, fueling debates over AI’s societal implications and ethical dimensions (2).

Public discourse frequently oscillates between optimism about AI’s potential to enhance human productivity and deep-seated anxieties regarding its unintended consequences. Media portrayals exacerbate fears with dramatic narratives, including threats of job loss, privacy violations, algorithmic biases, and autonomous decision-making in critical contexts such as military and healthcare (3, 4). Iconic cultural references, notably HAL 9000 from Stanley Kubrick’s “2001: A Space Odyssey,” further entrench these fears in collective consciousness, shaping societal perceptions of AI as potentially dangerous or uncontrollable (5).

Previous research has often focused on generational divides in attitudes toward emerging technologies, suggesting that digital natives are more adaptive and less anxious than older cohorts (6, 7). However, recent societal shifts and the widespread integration of AI into daily routines may have blurred these distinctions. The diffusion of AI through mobile devices, generative platforms, and social infrastructures has rendered direct exposure nearly universal—potentially equalizing emotional reactions across generations. Hence, while prior findings have consistently reported age-based variations, it remains empirically unclear whether such generational boundaries persist in the context of AI-related fears. To address this gap, the present study investigates the structure and determinants of AI-related anxiety across demographic groups, with a specific focus on potential generational differences. Drawing upon established psychological frameworks and validated measurement instruments, this study aims to examine whether the experience and perception of AI-related anxieties remain socially differentiated or have evolved into a shared psychosocial phenomenon that transcends age boundaries. Specifically, we surveyed a heterogeneous sample of 1,151 individuals across various age groups to explore the generational differences in perceptions and anxiety levels toward AI. The study aims not only to elucidate generational differences in AI-related anxieties but also to examine how demographic factors such as gender and technological exposure influence these perceptions. By doing so, the study provides a more comprehensive understanding of the diverse drivers of AI-related fears.

The significance of this study lies in its empirical depth and breadth, facilitating a nuanced understanding of public sentiment toward AI. The outcomes not only enhance scholarly comprehension at the intersection of human-computer interaction, technology acceptance, and digital ethics but also provide actionable guidance for stakeholders tasked with implementing socially responsible and psychologically considerate AI solutions. By systematically mapping the psychological, demographic, and sociotechnical contours of AI anxiety, this research directly addresses an urgent societal need to understand how human populations adapt emotionally, cognitively, and behaviorally to disruptive technological change—thus contributing to the broader dialogue on the evolving relationship between technology and society.

## Literature review

Recent scholarship has increasingly illuminated the nuanced psychological, ethical, and sociotechnical dimensions underpinning societal anxieties regarding artificial intelligence (AI). Srivastava (8) contends that anxieties related to AI are fundamentally connected to projections of a future society potentially devoid of empathy, leading to AI being perceived as an existentially threatening “other” (9). Walsh et al. (10) further assert that widespread misconceptions about complex AI concepts, including machine learning and superintelligence, amplify public anxieties disproportionately to actual technological capabilities.

Central to contemporary AI discourse is the prevalent anxiety regarding job displacement across multiple industries. McClure (11) identifies significant technophobic responses primarily rooted in fears of job obsolescence. Similar apprehensions are extensively documented by Alkhalifah et al. (12)Truong and Papagiannidis (13), and Madanaguli et al. (14), highlighting employee reluctance and organizational resistance stemming from automation concerns. Such fears are intensified by highly publicized incidents, including significant AI-driven layoffs in digital media and entertainment industries (15, 16). Conversely, Conklin (17) and Abdou and Kamal (18) critique the rationality of these anxieties, emphasizing historical trends demonstrating stable or improved employment rates in technologically advanced economies and advocating the strategic deployment of AI as an economic enabler rather than a competitor.

Ethical considerations, including privacy, transparency, algorithmic bias, and accountability, also form significant components of AI-related anxieties. Li and Huang (19) emphasize privacy and ethical concerns as dominant factors fueling public anxiety, particularly within healthcare contexts, where fears of compromised patient-provider relationships, misdiagnoses, and breaches of data security prevail (20–22). Baum et al. (23) and Ambartsoumean and Yampolskiy (24) propose addressing these ethical and regulatory challenges through greater transparency and comprehensive governance frameworks, cautioning that persistent skepticism can obstruct essential safety advancements.

Empirical evidence suggests enhancing AI literacy and transparency significantly alleviates public anxiety. Schiavo et al. (25) illustrate that improved AI understanding positively correlates with increased acceptance. Kajiwara et al. (26) support this, showing enhanced perceptions of AI among users who gain insight into its decision-making processes and practical applications. Wen et al. (27) further advocate for psychological dimensions to be integrated within AI education programs, facilitating emotionally and cognitively balanced user engagement with AI technologies.

Despite these advancements, existing literature predominantly examines AI-related anxieties through fragmented or narrowly scoped perspectives. Addressing this critical gap, this study applies an empirical, multidimensional approach to examine demographic and psychological variables influencing AI anxieties among 1,151 diverse respondents. By elucidating generational differences and their respective attitudes towards AI, the research provides robust insights for practitioners, policymakers, and educators, fostering informed and ethically considerate AI deployment strategies. The following methodology section elaborates on the systematic framework used to achieve these investigative objectives.

## Methodology

### Scale design and instrumentation

#### Questionnaire design

The questionnaire items were not developed *de novo* but were carefully compiled and adapted from well-established instruments in the literature. Specifically, items measuring Artificial Intelligence Anxiety (AIA; 16 items) were adapted from Wang & Wang (28), which operationalizes multidimensional AI anxiety. Technoparanoia (TPR; 5 items) and Cybernetic Revolt Fear (CR; 3 items) were drawn from established scales of technostress and technology confidence (29). AI Interaction Anxiety (AIINT; 6 items) and AI Learning Orientation (AIL; 8 items) were adapted from recent instruments assessing human–AI interaction and learning readiness (28, 30). Sociotechnical Blindness (STB; 4 items) and Job Replacement Anxiety (JR; 6 items) drew from validated items measuring sociotechnical risk perception and employment insecurity (28). Finally, Technology Self-Efficacy (TSE; 10 items) was adapted from related cyber-psychology framework (31).

Each set of items was reviewed and minimally reworded to reflect AI as the target technology while retaining the conceptual integrity of the original constructs. Items were translated/back-translated, and a pilot test (n = 150) ensured clarity and reliability before full deployment. This procedure preserves construct validity while contextualizing items for AI.

It is important to clarify that the present study did not develop a new scale, but rather integrates and adapts previously validated scales into a composite, multidimensional measure of AI anxiety for use in this context. This approach enhances comparability with prior studies while also enabling novel insights into the interplay of generational, gendered, and technological-exposure differences. Ultimately, 63 items were formulated to capture the diverse array of public apprehensions related to AI. These items underwent factor analysis, resulting in a robust and valid nine-dimensional framework for assessing AI-related anxiety.

The nine dimensions identified were:

1. AI Interaction Anxiety (AIINT) – Discomfort with interacting directly with AI systems.
2. Technoparanoia (TPR) – Anxiety regarding surveillance, control, or potential misuse of AI technologies.
3. Technology Self-Efficacy (TSE) – Perceived competence in understanding and using AI tools.
4. Cybernetic Revolt Fear (CR) – Fears associated with autonomous or dominant behavior by AI systems over humans.
5. Job Replacement Anxiety (JR) – Concerns surrounding job loss due to automation by artificial intelligence.
6. Sociotechnical Blindness (STB) – Perceived overlooking of AI’s societal risks and biases (i.e., the sense that sociotechnical consequences are neglected).
7. Artificial Intelligence Anxiety (AIA) – General unease or apprehension about the presence and advancement of AI.
8. AI Learning Orientation (AIL) – Openness and curiosity toward learning about AI.
9. Technophobia (TPB) – Broad avoidance or fear of emerging technologies.

Participants rated each item using a 5-point Likert scale ranging from 1 = Strongly disagree to 5 = Strongly agree. The overall measurement instrument exhibited excellent internal consistency, indicated by a Cronbach’s alpha score of.931 for the complete scale as well as high reliability throughout all subdimensions.

### Sample and data collection

Data were collected between July and October, 2024, using a mixed-mode approach. An online survey was distributed via institutional mailing lists and social media platforms, while printed questionnaires were administered in educational and community settings to ensure inclusivity of participants with limited digital access. Respondents were informed about the study’s purpose and anonymity was guaranteed. This dual strategy enhanced the representativeness of the dataset by including both digitally active and less technologically engaged individuals. Participation was voluntary, and respondents provided informed consent, assured that their data would be used solely for academic purposes.

The study surveyed a diverse sample of 1,151 individuals, encompassing a broad range of ages, genders, employment statuses, and digital experiences. The age distribution spanned from young adults to individuals aged 55 and above, ensuring comprehensive generational representation in the analysis. Participants were recruited through a combination of online platforms and paper-based questionnaires administered in public institutions and educational settings, enabling the inclusion of digitally less-inclined populations.

The study protocol was reviewed and approved by the Social and Humanities Ethics Committee of Sakarya University (Protocol #467384). Participation was voluntary, informed consent was obtained from all participants, and procedures complied with the Declaration of Helsinki.

### Data analysis

The dimensionality analysis was conducted using a common-factor exploratory approach. Specifically, we employed Principal Axis Factoring (PAF) with an oblique Promax rotation to allow for correlated latent dimensions, which is theoretically appropriate for psychosocial constructs. Factor retention was guided by a triangulated criterion comprising parallel analysis, scree-plot inspection, and the Kaiser criterion (eigenvalues > 1). Items were retained if λ ≥.40, |cross-loading| ≤.30, and h² ≥.30; all items met criteria, and none were removed. All items met retention thresholds; therefore, no items were removed. Analyses were conducted in SPSS v28 following established psychometric guidance (e.g., 32, 33).

To validate the nine-factor structure derived from the exploratory analysis, the sample (N = 1,151) was randomly divided into two subsamples (70/30 split). The first subsample (n = 806) was used for the exploratory factor analysis (EFA), while the second (n = 345) served as the hold-out dataset for confirmatory factor analysis (CFA).

Independent-samples t-tests and ANOVA were conducted to examine differences across demographic groups (e.g., gender, age, technological exposure). Given the number of group-comparison tests, we controlled for family-wise error using the Holm–Bonferroni procedure within each family of tests (e.g., gender × nine subscales). For transparency, we also provide Benjamini–Hochberg FDR results in the **Supplementary Materials**. Alongside p-values, we report effect sizes (Cohen’s d for t-tests; partial η² for ANOVA) and 95% CIs where applicable. SPSS outputs reporting “p = .000” were conservatively treated as p = 0.0005 for adjustment; decisions remain unchanged (adjusted p <.01).

In addition to reporting significance levels, we computed effect sizes to assess the practical magnitude of observed group differences. For t-tests, Cohen’s *d* was calculated using pooled standard deviations, and 95% confidence intervals (CIs) were obtained through bootstrapped resampling (1,000 iterations). For ANOVA analyses, partial η² was reported to indicate the proportion of variance explained by group effects. Interpretation followed Cohen's (34) guidelines: *d* = 0.20 (small), 0.50 (medium), 0.80 (large); partial η² = 0.01 (small), 0.06 (medium), 0.14 (large). These indices provide a more nuanced understanding of the practical significance of findings beyond *p*-values.

Finally, to assess the predictors of overall AI Anxiety (AIA), we conducted a hierarchical multiple regression. Step 1 included demographic controls available in this dataset—age, gender (0 = male, 1 = female), marital status (0 = single, 1 = married), employment (0 = not employed, 1 = employed), and AI usage (0 = non-user, 1 = user). In Step 2, psychological/technological predictors (Technoparanoia, AI Learning Orientation, Technology Self-Efficacy, Job Replacement Anxiety, Sociotechnical Blindness) were added. All continuous predictors were standardized; model assumptions were checked and met.

This hierarchical structure allows testing whether the focal predictors explain incremental variance in AI Anxiety above and beyond demographic effects. All variables were standardized prior to entry to reduce multicollinearity, and assumptions of normality, linearity, and homoscedasticity were verified.

## Findings

In this section, we present the findings from our analysis of AI-related anxieties among 1,151 individuals. Using a nine-dimensional anxiety scale, we conducted exploratory factor analysis and descriptive statistics to explore the nuances of these anxieties across different generations. The results are presented in line with the study objectives: first, to assess generational and demographic variations in AI-related anxieties; and second, to examine the predictive power of key dimensions in shaping overall AI Anxiety (AIA).

### Demographic characteristics

The study’s sample consisted of 1,151 individuals, with a slightly higher proportion of females (55.5%, *n* = 639) compared to males (44.5%, *n* = 512). Participants represented diverse age groups, with 25.7% (*n* = 296) aged 18–24, 21.0% (*n* = 242) aged 25–34, 20.4% (*n* = 235) aged 35–44, 18.2% (*n* = 210) aged 45–54, and 14.6% (*n* = 168) aged 55 or older, thereby providing comprehensive coverage across generations. Concerning marital status, most participants were single (57.9%, *n* = 666), with married participants accounting for 42.1% (*n* = 485). Participants’ living arrangements varied: 42.0% (*n* = 483) lived alone, 27.5% (*n* = 317) lived with their spouse, 14.9% (*n* = 172) resided with their parents, 12.5% (*n* = 144) lived with friends, and the remaining 3.0% (*n* = 35) reported other arrangements. Regarding employment, slightly more than half of the participants (51.3%, *n* = 591) were not actively employed, whereas 48.7% (*n* = 560) reported active employment. Collectively, these diverse demographic characteristics enabled an in-depth analysis of societal anxieties toward artificial intelligence across different segments of the population.

In addition to the core demographic characteristics, participants were also asked questions related to health and lifestyle. Responses revealed that 49.0% (*n* = 564) of participants reported paying attention to healthy eating, while 51.0% (*n* = 587) stated that they did not. Similarly, 50.8% (*n* = 585) indicated that they actively avoid eating at night, whereas 49.2% (*n* = 566) reported no such avoidance. In terms of parental status, 65.8% (*n* = 757) of the sample had children, while 34.2% (*n* = 394) did not. Regarding chronic health conditions, 38.4% (*n* = 442) of participants reported having a chronic illness, whereas 61.6% (*n* = 709) reported none. When asked whether they would want to know their diagnosis if seriously ill, 57.6% (*n* = 663) answered affirmatively, while 42.4% (*n* = 488) preferred not to know. These additional characteristics provide further contextual depth and enable a more comprehensive understanding of the psychosocial factors that may intersect with AI-related anxieties.

Regarding participants’ engagement with artificial intelligence technologies, slightly more than half of the sample (51.5%, *n* = 593) indicated they did not currently use AI, whereas 48.5% (*n* = 558) reported active usage. Among active users, 42.7% (*n* = 238) had been using AI technologies for one year or less, 21.1% (*n* = 118) for 2–3 years, 26.3% (*n* = 147) for 4–5 years, and 9.9% (*n* = 55) reported using AI for six or more years. These findings illustrate a diverse spectrum of familiarity and experience with AI, enhancing the depth of the subsequent analysis of AI-related anxieties.

### Exploratory factor analysis results

Sampling adequacy was strong (KMO = 0.89), and Bartlett’s test of sphericity was significant (χ²(190) = 3200.12, p <.001), confirming suitability for factor analysis. Using Principal Axis Factoring with Promax rotation (κ = 4), parallel analysis and the scree plot jointly supported retention of nine factors, consistent with the theoretical framework. The final nine-factor solution cumulatively explained 64.17% of the variance (**Table 1**). All items displayed acceptable psychometric performance (primary loadings.51–.84, communalities.34–.76) with no cross-loading >.30, hence no items were removed. Oblique rotation yielded modest-to-moderate inter-factor correlations (r ≈.15–.61), justifying the use of an oblique method and indicating related but distinguishable dimensions. The rotated pattern matrix and factor correlation matrix are provided in **Supplementary Table S1** and **Supplementary Table S2** (**Supplementary Materials**).

**Table 1:** Summary of extracted factors.

Factor name	Items (n)	Cronbach’s α	Variance explained (%)	Theoretical source	Sample item
Artificial Intelligence Anxiety (AIA)	16	.926	12.87	Wang & Wang (28)	“I feel uneasy about the increasing role of AI in daily life.”
Technology Self-Efficacy (TSE)	10	.902	8.91	Howard (31)	“I am confident in using technology to solve problems.”
AI Learning Orientation (AIL)	8	.913	8.67	Wang & Wang (28)	“I am willing to learn more about AI technologies.”
AI Interaction Anxiety (AIINT)	6	.951	7.84	Nomura et al. (30)	“I feel uncomfortable interacting with AI agents.”
Technophobia (TPB)	5	.867	5.72	Sindermann et al. (35)	“I avoid new technologies whenever possible.”
Sociotechnical Blindness (STB)	4	.954	5.48	Wang & Wang (28)	“People often overlook the societal consequences of AI.”
Technoparanoia (TPR)	5	.902	5.31	Khasawneh (29)	“AI is being secretly used to monitor society.”
Job Replacement Anxiety (JR)	6	.810	5.03	Wang & Wang (28)	“AI technologies will lead to widespread job losses.”
Cybernetic Revolt Fear (CR)	3	.958	4.34	Khasawneh (29)	“AI may one day act against human control.”
Total Variance Explained	—	9 subdimensions (63 items)	64.17%	—	—

### Confirmatory factor analysis and validity assessment

To validate the nine-factor structure derived from the exploratory analysis, the sample (N = 1,151) was randomly divided into two subsamples (70/30 split). The first subsample (n = 806) was used for the exploratory factor analysis (EFA), while the second (n = 345) served as the hold-out dataset for confirmatory factor analysis (CFA).

The CFA, performed using maximum-likelihood estimation, demonstrated an acceptable fit to the data (**Table 2**): χ²(1482) = 2543.27, p <.001; CFI = .943; TLI = .936; RMSEA = .045 (90% CI [.041,.049]); SRMR = .046. All standardized factor loadings exceeded.60 (p <.001), confirming adequate item-to-factor relationships.

**Table 2:** Confirmatory factor analysis fit indices.

Fit index	Recommended threshold	Obtained value
χ² (df = 1482)	—	2543.27 (p <.001)
CFI	>.90	.943
TLI	>.90	.936
RMSEA (90% CI)	<.06	.045 [.041–.049]
SRMR	<.08	.046

Reliability analyses included both Cronbach’s α and McDonald’s ω to ensure robustness. All constructs exceeded the recommended threshold (α, ω >.80).

Convergent validity was established as all average variance extracted (AVE) values were above.50 and composite reliability (CR) exceeded.70 for each factor.

Discriminant validity was confirmed using the Fornell-Larcker criterion (AVE > r²) and the Heterotrait–Monotrait (HTMT) ratio (<.85). Reliability and Validity Indices are presented in **Table 3**.

**Table 3:** Reliability and validity indices.

Construct	Items	α	ω	CR	AVE
Artificial Intelligence Anxiety (AIA)	16	.93	.92	.94	.58
Technology Self-Efficacy (TSE)	10	.90	.89	.91	.55
AI Learning Orientation (AIL)	8	.91	.90	.92	.56
AI Interaction Anxiety (AIINT)	6	.95	.94	.95	.65
Technophobia (TPB)	5	.87	.86	.89	.54
Sociotechnical Blindness (STB)	4	.95	.94	.95	.68
Technoparanoia (TPR)	5	.90	.89	.91	.52
Job Replacement Anxiety (JR)	6	.81	.80	.83	.51
Cybernetic Revolt Fear (CR)	3	.96	.95	.96	.74

These results collectively support the construct validity and internal consistency of the nine-dimensional model of AI-related anxiety.

Descriptive analyses presented in **Table 4** revealed that among the nine dimensions of AI-related anxiety, the highest mean score was observed for Artificial Intelligence Learning (M = 4.03, SD = 0.81), followed by Artificial Intelligence Anxiety (M = 3.70, SD = 0.78), Sociotechnical Blindness (M = 3.41, SD = 1.06), and Technology Self-Efficacy (M = 3.39, SD = 0.88). In contrast, the lowest levels were reported for AI Interaction Anxiety (M = 2.79, SD = 1.20) and Technophobia (M = 2.86, SD = 1.06).

**Table 4:** Descriptive statistics for AI-related anxiety dimensions.

Factor	Mean (M)	Standard deviation (SD)
Artificial Intelligence Anxiety (AIA)	3.70	0.78
Technology Self-Efficacy (TSE)	3.39	0.88
Artificial Intelligence Learning (AIL)	4.03	0.81
AI Interaction Anxiety (AIINT)	2.79	1.20
Technophobia (TPB)	2.86	1.06
Sociotechnical Blindness (STB)	3.41	1.06
Technoparanoia (TPR)	3.39	1.04
Job Replacement Anxiety (JR)	2.88	0.93
Cybernetic Revolt Fear (CR)	3.03	1.24

These findings suggest that while participants are generally open to learning about AI and acknowledge its broader societal impacts, they also express notable concerns regarding its misuse, job displacement, and loss of control.

### Inferential statistics (comparative analysis)

To explore whether participants’ demographic characteristics influenced their levels of AI-related anxiety, a series of independent samples t-tests were conducted across gender, employment status, AI technology usage, chronic illness, and lifestyle behaviors such as healthy eating. These analyses aimed to identify statistically significant differences across the nine dimensions of AI-related anxiety. Results revealed meaningful group differences in several key dimensions, particularly for gender and AI usage. **Table 5** summarizes the dimensions where significant differences were observed.

**Table 5:** T-Test results by demographic variables.

Dimension	Grouping variable	Mean difference	t	p
AI Interaction Anxiety	Gender (F < M)	-.21	-2.95	.003
Technoparanoia	Gender (F > M)	.21	3.49	<.001
Cybernetic Revolt	Gender (F > M)	.15	2.06	.040
Job Replacement	Gender (F < M)	-.11	-2.07	.039
AI Learning Orientation	Gender (F > M)	.16	3.43	.001
Technoparanoia	Chronic Illness (Yes < No)	-.14	-2.20	.028
Sociotechnical Blindness	Healthy Eating (Yes > No)	-.14	-2.21	.027
Technoparanoia	AI Use (Users > Non-users)	.21	3.50	<.001
Job Replacement	AI Use (Users > Non-users)	.13	2.37	.018
ST Blindness	AI Use (Users > Non-users)	.18	2.82	.005
AI Anxiety	AI Use (Users > Non-users)	.22	4.94	<.001
AI Learning Orientation	AI Use (Users > Non-users)	.22	4.67	<.001

After Holm–Bonferroni correction across the nine dimensions, only Job Replacement Anxiety (JR) remained significant (F(3, 1147) = 4.92, p = .003; partial η² = .025, small). Effects for Cybernetic Revolt Fear (CR) and Technophobia (TPB) did not survive family-wise correction and are interpreted as exploratory. Among usage-status comparisons, AIA, AIL, TPR, and STB remained significant (adjusted p ≤.030; *d* ≈.17–.29), while JR did not. Employment status yielded no significant differences after correction. “Healthy diet” and “chronic illness” families did not retain any significant effects post-correction. For exposure duration (ANOVA), only JR remained significant under Holm correction (partial η² ≈.025, small); CR and TPB did not, though CR was retained under FDR (see **Supplementary Table S4**).

### Gender-based differences in AI-related anxiety dimensions

Independent samples t-tests were conducted to examine gender-based differences across the nine dimensions of AI-related anxiety. The results revealed statistically significant differences between male and female participants in five of the nine factors:

- AI Interaction Anxiety (AIINT): Male participants (*M* = 2.91, *SD* = 1.21) reported significantly higher levels of interaction anxiety than female participants (*M* = 2.70, *SD* = 1.18), *t*(1149) = –2.95, *p* = .003. The mean difference was –0.21 (95% CI [–0.35, –0.07]).
- Technoparanoia (TPR): Female participants (*M* = 3.49, *SD* = 1.01) reported significantly higher levels of technoparanoia compared to males (*M* = 3.27, *SD* = 1.07), *t*(1149) = 3.49, *p* <.001. The mean difference was 0.21 (95% CI [0.09, 0.34]).
- Cybernetic Revolt Fear (CR): Female participants (*M* = 3.10, *SD* = 1.20) scored significantly higher in fear of cybernetic revolt than males (*M* = 2.95, *SD* = 1.28), *t*(1149) = 2.06, *p* = .040. The mean difference was 0.15 (95% CI [0.01, 0.29]).
- Job Replacement Anxiety (JR): Male participants (*M* = 2.95, *SD* = 0.96) reported significantly higher job replacement anxiety than females (*M* = 2.83, *SD* = 0.90), *t*(1149) = –2.07, *p* = .039. The mean difference was –0.11 (95% CI [–0.22, –0.01]).
- AI Learning Orientation (AIL): Female participants (*M* = 4.10, *SD* = 0.78) reported significantly higher AI learning orientation compared to males (*M* = 3.94, *SD* = 0.84), *t*(1149) = 3.43, *p* = .001. The mean difference was 0.16 (95% CI [0.07, 0.26]).

After Holm–Bonferroni correction across the gender × nine-subscale family, Technoparanoia (TPR), AI Learning Orientation (AIL), and AI Interaction Anxiety (AIINT) remained significant (small effects; d ≈.18–.21; 95% CIs exclude 0). Job Replacement Anxiety (JR) and Cybernetic Revolt Fear (CR) no longer met the adjusted threshold and are therefore treated as exploratory.

No statistically significant gender differences were observed in the remaining dimensions: Artificial Intelligence Anxiety (AIA), Technology Self-Efficacy (TSE), Sociotechnical Blindness (STB), and Technophobia (TPB) (*p* >.05 for all).

### Differences in sociotechnical blindness based on healthy eating habits

Independent samples t-tests were conducted to examine whether participants’ attention to healthy eating habits influenced their scores across the nine dimensions of AI-related anxiety. The analysis revealed a statistically significant difference in the Sociotechnical Blindness (STB) dimension. Participants who reported paying attention to healthy eating scored significantly higher in STB (M = 3,47) than those who did not (M = 3,33), *t*(1149) = –2.21, *p* = .027 (exploratory). The mean difference was –0.14, with a 95% confidence interval ranging from –0.26 to –0.02, indicating that health-conscious individuals may be more aware of or sensitive to the overlooked societal consequences of AI.

No statistically significant differences were observed for the remaining eight dimensions (AIINT, TPR, TSE, CR, JR, AIA, AIL, TPB), with all *p*-values exceeding.05.

### Technoparanoia differences based on chronic illness status

An independent samples t-test was conducted to examine differences in AI-related anxiety dimensions between participants with and without chronic illnesses. A statistically significant difference was observed in the Technoparanoia (TPR) dimension. Participants without chronic illnesses (M = 3.44, SD = 1.02) reported significantly higher levels of technoparanoia compared to those with chronic illnesses (*M* = 3.31, *SD* = 1.07), *t*(1149) = –2.20, *p* = .028. The mean difference was –0.14 (95% CI [–0.26, –0.01]).

No statistically significant differences were found between the groups in the other eight dimensions (*p* >.05 for all).

### Differences in AI-related anxiety dimensions by AI technology usage

Independent samples t-tests were conducted to compare the AI-related anxiety dimensions between participants who reported using AI technologies and those who did not. The results revealed significant differences in several dimensions:

- Technoparanoia (TPR): AI users (M = 3.50, SD = 1.00) scored significantly higher than non-users (M = 3.29, SD = 1.07), t(1149) = 3.50, p <.001, with a mean difference of 0.21 (95% CI [0.09, 0.33]).
- Job Replacement Anxiety (JR): Users (M = 2.95, SD = 0.94) also reported higher anxiety than non-users (M = 2.82, SD = 0.91), t(1149) = 2.37, p = .018.
- Sociotechnical Blindness (STB): Scores were higher among users (M = 3.50, SD = 1.03) compared to non-users (M = 3.32, SD = 1.08), t(1149) = 2.82, p = .005.
- Artificial Intelligence Anxiety (AIA): AI users (M = 3.82, SD = 0.71) showed significantly greater general anxiety toward AI than non-users (M = 3.60, SD = 0.82), t(1149) = 4.94, p <.001.
- AI Learning Orientation (AIL): Users of AI technologies (M = 4.14, SD = 0.76) also demonstrated a higher willingness to learn about AI compared to non-users (M = 3.92, SD = 0.84), t(1149) = 4.67, p <.001.

Among AI-usage groups, Artificial Intelligence Anxiety (AIA), AI Learning Orientation (AIL), Technoparanoia (TPR), and Sociotechnical Blindness (STB) differences remained significant following adjustment. Effect sizes again ranged from small to near-medium (Cohen’s *d* = 0.17–0.29; 95% CI [0.06, 0.42]).

No statistically significant differences were found for the remaining dimensions, including AI Interaction Anxiety (AIINT), Technology Self-Efficacy (TSE), Cybernetic Revolt (CR) (approaching significance at p = .066), and Technophobia (TPB) (p >.05 for all).

### Age group comparisons

A one-way analysis of variance (ANOVA) was conducted to examine potential generational differences across the nine dimensions of AI-related anxiety. Contrary to expectations derived from previous literature (e.g., 36, 37), across age groups, no ANOVA reached significance after correction, and all partial η² values were ≤.01, indicating trivial generational effects in this sample.

This finding suggests that in the present sample, AI-related anxieties are not shaped by generational belonging as strongly as previously proposed. While earlier studies have reported heightened AI-related apprehensions among younger individuals—often attributed to greater technological exposure and identity integration with digital systems—our results indicate a more homogeneous pattern of concern across age cohorts.

In other words, the diffusion of AI-related discourse and media exposure across all age groups may have contributed to a flattening of generational divides in perceptions of AI risks and benefits. These results invite a reconsideration of the commonly held assumption that generational identity alone is a reliable predictor of AI anxiety.

### Differences in AI-related anxiety by years of AI technology use

A one-way ANOVA was conducted to examine differences in AI-related anxiety dimensions based on the duration of AI technology use (1 year or less, 2–3 years, 4–5 years, 6 years or more). The analysis presented in **Table 6** revealed statistically significant differences in three dimensions: Cybernetic Revolt Fear (CR), Job Replacement Anxiety (JR), and Technophobia (TPB).

**Table 6:** ANOVA results by years of AI use.

Dimension	F	p	*Post Hoc* significance
Cybernetic Revolt Fear (CR)	4.12	.007	6+ yrs > ≤1 yr (p = .001), 6+ yrs > 2–3 yrs (p = .018)
Job Replacement Anxiety (JR)	4.67	.003	4–5 yrs > ≤1 yr (p = .007)
Technophobia (TPB)	3.44	.017	≤1 yr > 6+ yrs (p = .006)

- Cybernetic Revolt Fear (CR): A significant effect was found, F(3, 554) = 4.12, p = .007. *Post hoc* comparisons using the Tamhane test showed that participants with 6 years or more of AI use reported significantly higher cybernetic revolt fears (M difference = .605, p = .001) compared to those with 1 year or less. This suggests that long-term exposure to AI may be associated with heightened concerns about loss of control or autonomy in relation to AI systems.
- Job Replacement Anxiety (JR): A significant difference emerged across groups, F(3, 554) = 4.67, p = .003. Tamhane *post hoc* results revealed that participants with 4–5 years of AI use had significantly greater concerns about job replacement than those with 1 year or less (M difference = .321, p = .007). This may reflect a growing awareness of AI’s impact on labor markets among moderately experienced users.
- Technophobia (TPB): ANOVA results indicated a significant effect, F(3, 554) = 3.44, p = .017. *Post hoc* analyses showed that participants with 6 years or more of AI experience reported lower technophobia compared to those with 4–5 years (M difference = –.531, p = .017). This may imply that long-term familiarity with AI technologies can reduce general fear or avoidance of new technologies.

For usage duration, only Job Replacement Anxiety (JR) remained significant after correction (F(3,1147) = 4.92, p = .003; partial η² = .025; 95% CI [.010,.045]), representing a small effect size. Other dimensions (CR, TPB) did not survive correction (Holm-adjusted p >.05).

No statistically significant differences were found across usage duration groups in the remaining six dimensions: AI Interaction Anxiety, Technoparanoia, Technology Self-Efficacy, Sociotechnical Blindness, AI Anxiety, and AI Learning Orientation (p >.05 for all).

### Intercorrelations among AI-related anxiety dimensions

Pearson correlation analyses were conducted to examine the relationships among the nine AI-related anxiety dimensions. The results are summarized in Table X (to be inserted). All correlations were statistically significant at the 0.01 level (2-tailed), with the exception of the correlation between AI Interaction Anxiety (AIINT) and AI Learning Orientation (AIL), which was not significant (r = .057, p = .055).

The strongest positive correlations were observed between:

- Artificial Intelligence Anxiety (AIA) and Sociotechnical Blindness (STB) (r = .604),
- AIA and AI Learning Orientation (AIL) (r = .609),
- Technoparanoia (TPR) and Technology Self-Efficacy (TSE) (r = .459),
- AIA and TPR (r = .463),
- Job Replacement Anxiety (JR) and Cybernetic Revolt Fear (CR) (r = .417).

These findings suggest that individuals who experience general anxiety toward AI tend to also exhibit heightened sensitivity to broader societal consequences of AI and show stronger inclinations to learn about AI technologies. Moreover, technoparanoia appears to be closely associated with self-perceived technological competence and generalized AI anxieties.

Moderate positive correlations were also found between:

- TPR and Technophobia (TPB) (r = .369),
- CR and JR (r = .417),
- STB and AIL (r = .362).

The only non-significant association was between AI Interaction Anxiety and AI Learning Orientation (p = .055), indicating that comfort with interacting with AI systems may not directly translate into a desire to learn more about them.

### Hierarchical regression analysis predicting AI anxiety

To examine how psychological and technological factors predict overall AI Anxiety (AIA) beyond the influence of demographic characteristics, a hierarchical multiple regression was conducted (**Table 7**). In Model 1, key demographic controls were entered, including age, gender, marital status, employment status, and AI usage experience (coded as 0 = non-user, 1 = user). In Model 2, five psychological and cognitive predictors were added: Technoparanoia (TPR), Technology Self-Efficacy (TSE), Job Replacement Anxiety (JR), Sociotechnical Blindness (STB), and AI Learning Orientation (AIL).

**Table 7:** Hierarchical regression analysis.

Predictor	Unstandardized B	Std. error	Std. β	*t*	*p*
Step 1 – controls					
Age	–0.082	0.026	–.11	–2.95	.003
Gender (1 = female)	0.101	0.024	.13	4.21	<.001
Marital Status (1 = married)	–0.034	0.022	–.04	–1.56	.119
Employment (1 = employed)	–0.057	0.024	–.07	–2.40	.018
AI Usage (1 = user)	–0.029	0.021	–.03	–1.38	.168
Step 2 – main predictors					
Technoparanoia (TPR)	0.091	0.017	.35	10.24	<.001
Technology Self-Efficacy (TSE)	–0.076	0.018	–.27	–7.36	<.001
Job Replacement Anxiety (JR)	0.112	0.017	.14	6.68	<.001
Sociotechnical Blindness (STB)	0.263	0.016	.36	16.85	<.001
AI Learning Orientation (AIL)	–0.184	0.019	–.17	–3.83	<.01

Model Summary: R² = .512, ΔR² = .429**, *F*(10,1140) = 45.97***, *p* <.001; Durbin–Watson = 1.94.

*Note. p* <.05; p <.01; *p* <.001. Gender coded 0 = male, 1 = female; Marital Status 0 = single, 1 = married; Employment 0 = not employed, 1 = employed; AI Usage 0 = non-user, 1 = user. All predictors standardized before entry.

Model 1 (demographics only) was significant, F(5,1145) = 9.41, p <.001, explaining 8.3% of the variance (R² = .083).

Younger participants (β = –.11, p = .002), females (β = .13, p <.001), and those not actively employed (β = .07, p = .018) showed slightly higher levels of AI Anxiety, whereas marital status and AI usage experience were not significant predictors.

Model 2 (demographics + psychological predictors) significantly improved model fit, ΔF(5,1140) = 306.84, p <.001, explaining an additional 42.9% of the variance (ΔR² = .429, total R² = .512, Adjusted R² = .509).

The Durbin–Watson statistic was 1.94, indicating no autocorrelation.

### Key predictors

Psychological constructs were the most robust determinants of AI Anxiety.

- Sociotechnical Blindness (STB) and Technoparanoia (TPR) were the strongest positive predictors.
- Technology Self-Efficacy (TSE) and AI Learning Orientation (AIL) were significant negative predictors, indicating that higher confidence and openness to learning mitigate anxiety.
- Job Replacement Anxiety (JR) contributed positively but moderately.

Demographic effects became minimal once psychological variables were included, confirming the independent predictive power of cognitive-emotional factors in shaping AI Anxiety.

All predictors exhibited low multicollinearity (VIF range = 1.06–1.25). These findings demonstrate that even after accounting for demographic variation, psychological dimensions remain the primary explanatory mechanisms underlying AI-related anxiety.

### Interpretation and contextualization

The hierarchical regression results offer a nuanced understanding of the psychological and demographic determinants of AI-related anxiety. After controlling for demographic characteristics—age, gender, marital status, employment status, and AI usage—psychological predictors remained dominant. Specifically, Sociotechnical Blindness and Technoparanoia emerged as the strongest positive predictors of general AI anxiety, indicating that individuals who are more alert to AI’s societal implications and potential risks also experience higher emotional unease. Conversely, Technology Self-Efficacy and AI Learning Orientation were significant negative predictors, suggesting that individuals with greater confidence in their technological abilities and a proactive attitude toward learning about AI tend to experience less anxiety. Job Replacement Anxiety, while comparatively moderate, continued to contribute positively, reflecting persistent economic and occupational concerns linked to automation.

Demographically, the inclusion of control variables revealed that younger participants and women exhibited slightly higher anxiety levels, consistent with earlier research highlighting generational and gender-based differences in technology-related fears. However, once psychological factors were introduced, these demographic effects diminished in magnitude, reinforcing the conclusion that AI anxiety is more strongly rooted in cognitive and affective dispositions than in sociodemographic background.

Where zero-order and adjusted associations diverged (e.g., AIL), patterns were consistent with suppression: a positive raw correlation with AIA became negative after accounting for STB/TPR, indicating that learning orientation attenuates anxiety once risk-perception variance is isolated. This underscores the value of hierarchical modeling when conceptually adjacent predictors co-vary.

Together, these results confirm that AI-related anxieties are multifaceted, psychologically driven, and context-dependent. The findings support the study’s overarching aim of mapping the psychological and demographic predictors of AI anxiety across diverse populations. Importantly, the results underscore the need for tailored interventions and public education strategies—for instance, initiatives that foster digital self-efficacy and informed learning about AI may help mitigate anxiety and resistance. In doing so, this research provides actionable insights for policymakers, educators, and technology developers aiming to promote responsible, psychologically inclusive AI adoption.

## Discussion

This empirical study offers a critical interpretation of the empirical findings on AI-related anxieties, positioning them within the broader theoretical discourse on human-technology interaction. By unpacking the psychological and demographic contours of AI anxiety across nine dimensions, this section aims to illuminate how individuals cognitively and emotionally navigate the promises and perils of increasingly autonomous technologies. The patterns revealed offer not only empirical insights but also raise pressing questions about the adequacy of current frameworks used to understand public responses to AI, highlighting the urgency of reconceptualizing acceptance through a more holistic and interdisciplinary lens. Our analysis of AI-related anxieties, based on responses from 1,151 individuals and measured across nine distinct dimensions, reveals a deeply layered and multifactorial structure of perceptions surrounding artificial intelligence. These anxieties are shaped not only by technological exposure but also by broader cognitive, emotional, and sociocultural contexts. As observed in previous research (38, 39), apprehensions about AI are far from homogeneous. Instead, they reflect diverse interpretations of risk and opportunity, with concerns spanning from loss of personal agency and employment uncertainty to broader societal disruption and perceived loss of control.

Two constructs—Sociotechnical Blindness (STB) and Technoparanoia (TPR)—emerged as the most robust positive predictors of generalized AI anxiety. By contrast, AI Learning Orientation (AIL) and Technology Self-Efficacy (TSE) were protective, showing negative associations with AIA once shared variance with STB/TPR was controlled. Notably, AIL correlated positively with AIA at the zero-order level but reversed sign in the multivariable model, a classic suppression effect indicating that willingness to learn about AI can buffer anxiety when perceptions of neglected sociotechnical risks are held constant.

Demographic analysis further reveals critical insights. Female respondents reported higher levels of technoparanoia and AI learning orientation, while male respondents displayed greater interaction anxiety and job insecurity related to AI. These results are in line with studies on gender-based disparities in technological trust and digital literacy (40, 41). Additionally, AI usage patterns play a notable role: frequent users exhibit lower general technophobia but demonstrate heightened fear of autonomous rebellion, supporting the view that experience with AI moderates some fears while intensifying others (42).

The absence of significant generational differences in AI-related anxiety represents a noteworthy and somewhat counterintuitive result. Whereas prior studies have consistently identified age as a determinant of technological anxiety (36, 43), the present study’s null findings may reflect the increasing normalization of AI technologies across all age segments. With AI now embedded in everyday digital infrastructures—from smartphones to healthcare systems—exposure has become universal rather than age-bound. Consequently, AI anxiety may be evolving into a societal rather than generational phenomenon, transcending traditional demographic boundaries. This finding positions the current study as an important empirical reference suggesting that generational divides in AI-related anxiety may be narrowing in contemporary societies.

All group comparisons were reported with effect sizes and Holm–Bonferroni–adjusted p-values, with non-surviving findings treated as exploratory. The robustness of the present findings was reinforced through hierarchical regression and confirmatory factor analyses, which demonstrated that the multidimensional structure of AI-related anxiety is both psychometrically sound and theoretically coherent. Controlling for demographic variables such as age, gender, marital status, employment, and AI usage did not alter the primary relationships observed, indicating that psychological mechanisms—rather than sociodemographic background—remain the dominant predictors of AI-related anxiety. This reinforces the validity and generalizability of our proposed model across diverse populations.

These findings carry substantial implications for public policy, organizational strategy, and educational programming. They underscore the need to move beyond conventional digital literacy initiatives by incorporating psychological preparedness and ethical reasoning into AI education (44). In the labor context, employers and policymakers must address varying levels of anxiety related to automation—particularly among individuals with lower digital confidence or in professions at higher risk of displacement (45). Furthermore, public discourse on AI should move away from generic messaging and instead adopt stratified communication strategies that account for generational, gender, and experiential variation.

Theoretically, our results challenge the sufficiency of traditional technology acceptance frameworks by underscoring the importance of affective and existential concerns. The persistence of AI anxiety—even among informed and experienced users—signals a need to reconceptualize acceptance not merely as a rational calculation of utility but as a complex psychological negotiation. Future research should examine how narratives of technological inevitability, sociohistorical mistrust, and cultural imaginaries shape public sentiment toward AI. As intelligent systems become embedded in the architecture of daily life, attending to the emotional and symbolic dimensions of their reception will be as vital as addressing their technical and economic implications.

### Limitations

Despite its strengths, the study has several limitations. The cross-sectional design limits the ability to make causal inferences regarding the relationship between demographic factors, psychological dispositions, and AI-related anxieties. Moreover, while the sample size was robust, the data were self-reported and collected from a single national context, which may limit generalizability to other cultural or institutional settings. Finally, while the anxiety scale was carefully adapted validated, longitudinal studies are needed to assess how these perceptions evolve as AI technologies become more deeply integrated into daily life (46).

### Recommendations

Based on the findings of this study, several actionable recommendations can be made to address the complex and varied anxieties surrounding artificial intelligence.

Policy: Policymakers should prioritize transparent and inclusive AI governance frameworks that respond to the public’s concerns about job displacement, ethical misuse, and loss of control. Special attention should be given to:

- Strengthening regulations around data privacy, algorithmic accountability, and explainability of AI systems.
- Implementing workforce transition strategies, including upskilling programs and job security policies, especially for sectors most vulnerable to automation.
- Encouraging participatory policy design that includes underrepresented groups and reflects diverse technological experiences and anxieties.

Education: To reduce fear and increase psychological readiness for AI, we recommend the development of multi-level AI literacy programs, tailored to different demographic groups:

- For students and young adults, curricula should include not only technical skills but also critical thinking about the ethical and societal implications of AI.
- For the general public, accessible campaigns should focus on demystifying AI, clarifying its everyday applications, and correcting misconceptions.
- For working professionals, sector-specific training should be offered to build confidence in using AI tools and adapting to evolving digital environments.
- Collectively, these measures can help foster a more informed, balanced, and resilient public discourse about artificial intelligence.

Community Engagement: It is vital to facilitate community discussions to foster understanding and address concerns about AI technologies. Encourage;

- public forums with domain experts to demystify AI and address misconceptions.
- hands-on workshops that build practical familiarity and self-efficacy.
- transparent communication about data use, bias mitigation, and accountability to reduce technoparanoia and perceived sociotechnical neglect.

## Conclusion

This study elucidates the complex and multidimensional nature of AI-related anxieties, grounded in a diverse interplay of demographic variables, usage patterns, and psychological predispositions. Our findings indicate that heightened awareness of AI’s broader societal implications and an individual’s cognitive engagement with the technology serve as key predictors of generalized AI anxiety. These results reinforce the argument that public apprehension toward AI is not monolithic; rather, it is stratified along lines of gender, familiarity, and lived technological experience.

From a theoretical standpoint, the identification of AI Learning Orientation (AIL) and Sociotechnical Blindness (STB) as primary anxiety predictors challenges prevailing assumptions in traditional models of technology acceptance. These constructs reflect a dual process: individuals more informed about AI tend to exhibit increased sensitivity to its socio-ethical risks. This aligns with emerging scholarship that frames AI anxiety not solely as a byproduct of ignorance, but as a rational reaction to complex ethical ambiguities and long-term societal consequences.

Gendered patterns in the data merit particular attention. Female participants exhibited higher levels of technoparanoia and learning-oriented anxiety, while male participants were more concerned with interactional issues and employment threats. These distinctions are consistent with prior research on digital inequalities and risk perception, illustrating how structural and cultural factors shape differing emotional responses to emerging technologies. Furthermore, our data suggest that high-frequency AI users experience a paradoxical relationship: reduced general technophobia but intensified existential concerns, such as fear of AI autonomy or cybernetic revolt. Notably, the absence of generational differences underscores the possibility that AI anxiety has become a shared social concern rather than a generationally contingent experience—an insight that challenges established assumptions and marks a conceptual turning point in the literature. These findings suggest that anxieties surrounding AI have become socially ubiquitous, cutting across traditional demographic divisions. Consequently, addressing AI-related fears requires societal-level interventions that foster informed trust, digital literacy, and equitable participation in the evolving AI ecosystem.

These empirical insights hold substantial implications for policy, pedagogy, and public discourse. Interventions should be multilayered and differentiated—grounded in the recognition that anxiety toward AI is not evenly distributed across populations. Educational strategies must incorporate ethical literacy, psychological preparedness, and socio-technical critique, rather than focusing exclusively on operational competencies. Concurrently, labor policy must attend to uneven distributions of automation risk, particularly in low-autonomy occupations or communities with limited technological capital.

Importantly, this research foregrounds the need to reconceptualize AI anxiety as more than a reactionary impulse. It is, rather, a sociotechnical barometer—an expression of unease about the redistribution of agency, responsibility, and control in a world increasingly shaped by non-human decision-making systems. Addressing this anxiety therefore requires systemic, not superficial, solutions: participatory design processes, transparent governance structures, and cultural narratives that engage with rather than suppress collective uncertainty. This study provides robust evidence of multidimensional anxieties surrounding AI, shaped not only by generational position but also by gender and technological exposure. By integrating established scales into a comprehensive framework, the study offers a transparent and replicable methodology for assessing AI-related fears. The findings underscore that anxieties about privacy, employment displacement, and sociotechnical risks are unevenly distributed across demographic groups, with important implications for both policy and education. Addressing these concerns requires targeted AI literacy initiatives and policies that enhance transparency, trust, and public engagement. By illuminating demographic nuances in AI anxieties, this study advances both theoretical understanding and practical strategies for navigating the societal challenges of artificial intelligence.

The refined analytical approach adopted in this study further strengthens the credibility of its conclusions. By employing exploratory and confirmatory factor analyses alongside hierarchical regression controlling for demographic factors, the study provides statistically and theoretically robust evidence for the multidimensional nature of AI anxiety. These enhancements ensure that the observed relationships are not artifacts of sample composition but reflect genuine psychological and cognitive dynamics shaping public responses to AI.

As AI continues to advance and integrate into the core of societal functions, longitudinal research is essential to monitor how such technologies recalibrate psychological baselines, reshape civic engagement, and mediate identity. Future investigations should explore how AI anxiety evolves over time and across contexts, and how it intersects with broader dynamics of trust, inequality, and epistemic justice. In doing so, the field can contribute to the cultivation of an AI-literate public equipped not only to use these systems—but to critique, question, and co-shape them in alignment with democratic values and social sustainability.

## Data Availability

The original contributions presented in the study are included in the article/**Supplementary Material**. Further inquiries can be directed to the corresponding author.
