# Supplementary material for: The social anatomy of AI anxiety: gender, generations, and technological exposure
Source: Front Psychiatry. 2025 Nov 21;16:1641546. doi: 10.3389/fpsyt.2025.1641546 (PMC12679909; doi:10.3389/fpsyt.2025.1641546)
Supplement: Supplementary file 1 [file Supplementaryfile1.docx]

**Table S1. Rotated Pattern Matrix (Principal Axis Factoring, Promax Rotation)**

| **Item Code** | **Factor 1: AIINT** | **Factor 2: TPR** | **Factor 3: TSE** | **Factor 4: CR** | **Factor 5: JR** | **Factor 6: STB** | **Factor 7: AIA** | **Factor 8: AIL** | **Factor 9: TPB** | **Communality** |
| --- | --- | --- | --- | --- | --- | --- | --- | --- | --- | --- |
| AIINT1 | .78 | .09 | .12 | .05 | .10 | .06 | .03 | .04 | .08 | .64 |
| AIINT2 | .73 | .11 | .08 | .09 | .07 | .10 | .05 | .06 | .04 | .59 |
| TPR1 | .10 | .83 | .09 | .06 | .07 | .05 | .03 | .02 | .05 | .68 |
| TPR2 | .08 | .79 | .10 | .04 | .09 | .07 | .06 | .05 | .04 | .64 |
| TSE1 | .11 | .09 | .77 | .06 | .08 | .04 | .09 | .07 | .05 | .61 |
| TSE2 | .07 | .08 | .74 | .09 | .06 | .10 | .04 | .08 | .05 | .58 |
| CR1 | .09 | .06 | .08 | .82 | .05 | .07 | .05 | .04 | .03 | .67 |
| CR2 | .10 | .07 | .06 | .79 | .07 | .09 | .06 | .05 | .05 | .63 |
| JR1 | .08 | .07 | .05 | .06 | .81 | .10 | .07 | .04 | .03 | .65 |
| JR2 | .09 | .06 | .09 | .05 | .76 | .11 | .04 | .05 | .06 | .60 |
| STB1 | .10 | .09 | .08 | .07 | .06 | .80 | .09 | .04 | .05 | .67 |
| STB2 | .11 | .10 | .09 | .05 | .08 | .76 | .06 | .05 | .07 | .63 |
| AIA1 | .06 | .04 | .07 | .05 | .09 | .10 | .82 | .08 | .06 | .68 |
| AIA2 | .08 | .06 | .05 | .09 | .08 | .06 | .78 | .07 | .05 | .64 |
| AIL1 | .09 | .05 | .08 | .07 | .06 | .05 | .08 | .81 | .05 | .69 |
| AIL2 | .07 | .07 | .06 | .09 | .08 | .06 | .07 | .77 | .04 | .64 |
| TPB1 | .08 | .06 | .07 | .05 | .06 | .08 | .06 | .04 | .80 | .67 |
| TPB2 | .06 | .05 | .09 | .07 | .09 | .06 | .08 | .05 | .76 | .62 |

**Extraction method:** Principal Axis Factoring
**Rotation method:** Promax with Kaiser normalization
**Loadings below .30 are suppressed.**
**Cumulative variance explained:** 64.76%

**Table S2. Factor Correlation Matrix (Oblique Promax Rotation)**

| **Factors** | **1** | **2** | **3** | **4** | **5** | **6** | **7** | **8** | **9** |
| --- | --- | --- | --- | --- | --- | --- | --- | --- | --- |
| **1. AIINT** | 1.00 | .29 | .31 | .22 | .36 | .25 | .18 | .21 | .23 |
| **2. TPR** | .29 | 1.00 | .38 | .27 | .33 | .19 | .22 | .20 | .26 |
| **3. TSE** | .31 | .38 | 1.00 | .25 | .21 | .24 | .27 | .28 | .22 |
| **4. CR** | .22 | .27 | .25 | 1.00 | .31 | .26 | .23 | .20 | .24 |
| **5. JR** | .36 | .33 | .21 | .31 | 1.00 | .34 | .30 | .25 | .29 |
| **6. STB** | .25 | .19 | .24 | .26 | .34 | 1.00 | .32 | .27 | .21 |
| **7. AIA** | .18 | .22 | .27 | .23 | .30 | .32 | 1.00 | .39 | .26 |
| **8. AIL** | .21 | .20 | .28 | .20 | .25 | .27 | .39 | 1.00 | .33 |
| **9. TPB** | .23 | .26 | .22 | .24 | .29 | .21 | .26 | .33 | 1.00 |

**Note:** All correlations are significant at *p* < .001.
Inter-factor correlations ranged from **.15 to .61**, confirming that constructs are related but distinct — validating the choice of oblique rotation.

**Table S3. Latent Factor Correlation Matrix (CFA Model)**

|  | **AIA** | **TSE** | **AIL** | **AIINT** | **TPB** | **STB** | **TPR** | **JR** | **CR** |
| --- | --- | --- | --- | --- | --- | --- | --- | --- | --- |
| AIA | — | .38 | .42 | .55 | .29 | .41 | .46 | .32 | .30 |
| TSE | — | — | .47 | .33 | .21 | .27 | .18 | .25 | .19 |
| AIL | — | — | — | .35 | .26 | .30 | .23 | .28 | .22 |
| AIINT | — | — | — | — | .36 | .42 | .44 | .40 | .35 |
| TPB | — | — | — | — | — | .26 | .30 | .24 | .20 |
| STB | — | — | — | — | — | — | .36 | .34 | .28 |
| TPR | — | — | — | — | — | — | — | .41 | .37 |
| JR | — | — | — | — | — | — | — | — | .26 |
| CR | — | — | — | — | — | — | — | — | — |

**Table S4. Holm-adjusted p-values and effect sizes by family**

**Gender (t-tests; m=9)**

| **Factor** | **p (raw)** | **p (Holm-adj)** | **Cohen’s d** | **Result** |
| --- | --- | --- | --- | --- |
| **TPR** | < .001 | **0.0045** | **0.21** | **Sig.** |
| **AIL** | .001 | **0.0080** | **0.20** | **Sig.** |
| **AIINT** | .003 | **0.0210** | **0.18** | **Sig.** |
| JR | .039 | 0.234 | 0.12 | n.s. |
| CR | .040 | 0.234 | 0.12 | n.s. |
| AIA | .073 | 0.292 | — | n.s. |
| TPB | .091 | 0.292 | — | n.s. |
| STB | .506 | 1.000 | — | n.s. |
| TSE | .740 | 1.000 | — | n.s. |

***AI Technology Usage*** (t-tests; m=9)

| **Factor** | **p (raw)** | **p (Holm-adj)** | **Cohen’s d** | **Result** |
| --- | --- | --- | --- | --- |
| **AIA** | < .001 | **0.0045** | **0.29** | **Sig.** |
| **AIL** | < .001 | **0.0045** | **0.28** | **Sig.** |
| **TPR** | < .001 | **0.0045** | **0.21** | **Sig.** |
| **STB** | .005 | **0.030** | **0.17** | **Sig.** |
| JR | .018 | 0.090 | 0.12 | n.s. |
| CR | .066 | 0.264 | — | n.s. |
| AIINT | .221 | 0.663 | — | n.s. |
| TSE | .272 | 0.663 | — | n.s. |
| TPB | .444 | 0.663 | — | n.s. |

**Employment status (t-tests; m=9)**

All adjusted p ≥ .90 → **n.s**

**Healthy diet (t-tests; m=9)**

| **Factor** | **p (raw)** | **p (Holm-adj)** | **Result** |
| --- | --- | --- | --- |
| STB | .027 | 0.243 | n.s. |
| Others p > .05 and adjusted p ≥ .51 → **n.s.** |  |  |  |

**Chronic illness (t-tests; m=9)**

| **Factor** | **p (raw)** | **p (Holm-adj)** | **Result** |
| --- | --- | --- | --- |
| TPR | .028 | 0.252 | n.s. |
| Others p > .05 and adjusted p ≥ .59 → **n.s.** |  |  |  |

**Years of AI Use (ANOVA; m=9)**

| **Factor** | **p (raw)** | **p (Holm-adj)** | **partial η²** | **Result** |
| --- | --- | --- | --- | --- |
| **JR** | .003 | **0.027** | **.025** | **Sig.** |
| CR | .007 | 0.056 | .022 | n.s. (Holm) |
| TPB | .017 | 0.119 | .018 | n.s. |
| Others | > .05 | ≥ .336 | — | n.s. |

**Table S5. Adjusted p-values, Effect Sizes, and Confidence Intervals**

| **Comparison** | **Factor** | **Adjusted p** | **Effect Size** | **95% CI** | **Interpretation** |
| --- | --- | --- | --- | --- | --- |
| Gender | Technoparanoia (TPR) | **.0045** | *d* = 0.21 | [0.09, 0.33] | Small |
| Gender | AI Learning Orientation (AIL) | **.0080** | *d* = 0.20 | [0.08, 0.31] | Small |
| Gender | AI Interaction Anxiety (AIINT) | **.0210** | *d* = 0.18 | [0.06, 0.29] | Small |
| AI Use | Artificial Intelligence Anxiety (AIA) | **.0045** | *d* = 0.29 | [0.17, 0.40] | Small–Medium |
| AI Use | AI Learning Orientation (AIL) | **.0045** | *d* = 0.28 | [0.16, 0.39] | Small–Medium |
| AI Use | Technoparanoia (TPR) | **.0045** | *d* = 0.21 | [0.09, 0.33] | Small |
| AI Use | Sociotechnical Blindness (STB) | **.030** | *d* = 0.17 | [0.05, 0.29] | Small |
| AI Exposure Duration | Job Replacement Anxiety (JR) | **.027** | partial η² = .025 | [.010, .045] | Small |
